# “Ego massaging that helps”: a framework analysis study of internal medicine trainees’ interprofessional collaboration approaches

**DOI:** 10.1080/10872981.2023.2243694

**Published:** 2023-08-03

**Authors:** Joanne Kerins, Samantha Eve Smith, Victoria Ruth Tallentire

**Affiliations:** a Scottish Centre for Simulation and Clinical Human Factors, NHS Forth Valley, Larbert, UK; b Acute medicine, NHS Greater Glasgow and Clyde, Glasgow, Scotland, UK; c Medical Education Directorate, NHS Lothian, Edinburgh, UK; d Medical Directorate, NHS Education for Scotland, Edinburgh, Scotland, UK; e College of Medicine and Vetinary Medicine, University of Edinburgh, Edinburgh, Scotland, UK

**Keywords:** Interprofessional collaboration, internal medicine, continuing professional development, framework analysis, qualitative research

## Abstract

**Introduction:**

Patient care depends on collaborative practice. Debate remains as to the best approach to providing education for collaboration, with educational interventions often far removed from the realities of the clinical workplace. Understanding the approaches used for collaboration in clinical practice could inform practical strategies for training. For internal medicine trainees, this involves collaboration with other professions but also with other specialties. This study aimed to explore the approaches that internal medicine trainees use for interprofessional collaboration and the ways that these approaches vary when internal medicine trainees interact with different healthcare provider groups.

**Methods:**

Following ethical approval and participant consent, interprofessional communication workshops between August 2020 and March 2021 were audio recorded and transcribed verbatim. Workshops involved groups of internal medicine trainees discussing collaboration challenges and the approaches they use in clinical practice. This framework analysis study used the interprofessional collaboration framework described by Bainbridge and Regehr (building social capital, perspective taking and negotiating priorities and resources), and cross-referenced the categorised data with the healthcare groups that trainees collaborate with, to look for patterns in the data.

**Results:**

Seventeen workshops, involving 100 trainees, were included. Trainees described relationship building, perspective taking and negotiating priorities and resources. Relationship building was a modification to the original framework domain of building social capital. Themes of power and civility transcended domains with evidence of using hierarchy as leverage when negotiating and employing civility as a tactical approach throughout.

**Discussion:**

This bi-dimensional analysis highlights patterns of perspective taking when collaborating with other specialties and professions, and the approaches to negotiation of courting favour and coercion when interacting with other specialties. This study provides evidence of the strategies currently utilised by internal medicine trainees, with different healthcare groups, and presents a modified framework which could inform the development of training for collaboration.

## Introduction

Interprofessional collaboration (IPC) is crucial for the effective delivery of healthcare [1–3.] IPC ‘enables shared knowledge and skills of health care providers to synergistically influence the patient care provided’ [4]. Debate remains as to how best to prepare healthcare professionals for IPC [5]. The complex social interplay between healthcare provider groups can present obstacles to collaborative practice [6–10]. In particular, collaborations between medical trainees and their colleagues can be challenging [11,12]. Inter-specialty conflict continues to afflict the medical profession [13–17] and role dissonance can impede effective collaboration [11]. Although efforts have been made to explore collaboration within internal medicine in the simulated setting [18], there is a need to better understand how trainees collaborate within the clinical workplace.

Interprofessional education (IPE) aims to reflect the multidisciplinary nature of the clinical environment and promote effective collaboration [19–22]. However, the discrepancy between the often idealistic IPE context and the realities of the clinical domain could negate its benefits [5,13,23,24]. Furthermore, there remains limited evidence of the impact of IPE interventions on behaviour change and organisational practice [25,26]. In response to concerns around IPE, the concept of ‘education for collaboration’ [5] widens the outlook on potential strategies, with calls to incorporate training within the clinical workplace [20]. Whilst this is a step closer to representing clinical practice, a deeper insight into what collaboration looks like in reality is lacking.

Although IPE often takes place at the undergraduate level, postgraduate trainees possess an entrenched understanding of workplace roles and system issues contributing to collaborative care [5]. Different professions approach collaboration in different ways [27] and examining these could advance our understanding of collaboration in practice. For the purposes of this study, IPC will refer to dynamics between groups, including those from different professions and those from different specialties or grades within a specialty. We aim to link theory with clinical practice to investigate which approaches are utilised and in what circumstances they may help.

### Conceptual framework

This study uses the framework for interprofessional collaboration (IPC) outlined by Bainbridge and Regehr in 2015 as its conceptual backbone [23]. Bainbridge and Regehr argue that ‘individual ways of thinking’ should be considered to promote IPC, shifting the focus from team behaviours [23]. Four domains are included for training in IPC: building social capital; perspective taking; negotiating priorities and resources; and conflict management. To date, there has been little empirical testing of how such individual approaches to developing collaborative networks might translate to the workplace. Alternative perspectives on IPC, such as competency frameworks, have been developed including domains such as communication and teamwork [28]. However, how to succeed in these domains, and specifically how to train healthcare professionals to succeed, remains elusive. Bainbridge and Regehr’s framework was chosen for this study due to its tangible approaches to collaboration in practice, such as negotiation strategies [23].

Power differentials have been recognised as key in group dynamics [6] but issues of power have been somewhat neglected in relation to IPE in the past. This is a particularly pertinent issue between healthcare provider groups. How best to train health professionals to deal with these issues remains a priority [5,29]. They are inherent within workplace systems and Bainbridge and Regehr’s framework provides a useful starting point for exploration [23].

This study has two aims:

1. Exploring the approaches that internal medicine trainees use for IPC.
2. Exploring the ways that these approaches vary when internal medicine trainees interact with different healthcare provider groups.

## Materials and methods

### Ethical approval

NHS Education for Scotland ethics review board granted ethical approval for this study, reference number NES/Res/14/20/Med. All participants gave written consent for data collection and the publication of anonymised results.

### Context

Internal medicine training is a three-year training programme for doctors in the United Kingdom (UK) who wish to pursue a career in hospital medicine. Between August 2020 and March 2021, 124 internal medicine trainees participated in an interprofessional communication workshop in groups of six. The workshop, attended only by internal medicine trainees, was on the topic of interprofessional communication. It has been argued that education for collaboration should provide uni-professional opportunities which can address workplace structures, power and conflict, of which this workshop is an example [5]. Key learning objectives were to explore challenges of interprofessional interactions and collaboration approaches. The workshop was facilitated by two consultant physicians who aimed to create a safe space for trainee-led discussion. The discussion was guided by participants with trainees setting their own agenda at the start of the session. Trainees were asked to voice areas of difficulty regarding interprofessional interactions and a facilitator documented these on a paper flipchart. Thereafter, a free-flowing discussion followed this agenda. Facilitators used open questions to enquire about experiences and prompt reflection on the impact of challenges and the strategies that trainees have employed.

### Data collection

Workshops in which all participants had given their consent were audio recorded. This approach was chosen to obtain participants’ descriptions of encounters in the clinical workplace, whilst not influencing their dialogue or learning experience [30]. The approaches described by Bainbridge and Regehr were not familiar to the facilitators [23], and so the discussions were not led in the direction of any particular strategies. Instead, the discussions aimed to provide a realistic and uninfluenced representation of internal medicine trainees’ reflections on their behaviour in the clinical workplace. JK was present at all workshops, as a non-participating researcher, to record the discussion and become immersed in the data. Audio recordings of workshops were anonymised and transcribed verbatim.

### Data analysis

Framework analysis is a qualitative research approach, first developed in social policy research, which aims to generate actionable outcomes [31]. The Bainbridge and Regehr framework is displayed with definitions and real world examples in in Table 1 [23]. We excluded the conflict management domain (present in the original framework) for this study, in order to focus on strategies used for collaboration and thereby prevention of conflict [23]. Framework analysis provides the opportunity to compare and contrast data across cases, or in this context, healthcare groups in the workplace [35]. It involves the creation of a matrix structure, which provides a visual representation, allowing researchers to appreciate patterns in the data [31,35].

**Table 1.** Domains of IPC with definitions as described by Bainbridge and Regehr [23].

Domain	Descriptions	Illustrative example	Context specific example
Building social capital	- Straight exchange as found in other forms of capital [23] - Goodwill available to individuals or groups due to social relations [32]	Taking out a neighbour’s bin for them as they have done the same for you	Buying food to share with ward team during nightshift
Perspective taking	- Checking what others know or perceive [33] - Imagining what the point of view of the other person or people may be [23]	Considering the perspective of someone from a different political persuasion	Bearing in mind the perspective of allied health professionals when referring patients, relating to the information they need
Negotiating priorities and resources	- Process of persuasion or influence - **Consensus building**- accommodate and manage different goals and priorities effectively [23] - **Influencing strategies** people use at work [34]: **Reason**- ‘Using reason, justification and logic to make a request’ [34] **Assertion**- ‘Making a direct request for what we want and how we feel about the situation, including persisting with requests’[34] **Courting favour**- ‘Bringing oneself into favour with the other person by being friendly to them or positive about them’[34] **Coercion**- ‘Threatening to use, or actually using, some sort of sanction, including being unco-operative and doing as you wish’ [34] **Partnership**- ‘Getting the support of others at all levels both within and outside the immediate situation’ [34]	- Siblings discussing and agreeing together how to spend a family holiday (*consensus building*) - Threatening to buy a car elsewhere if price not reduced when negotiating with car salesperson (*coercion*)	- Explaining the need for an urgent portable chest. x-ray due to patient condition (*reason*) Insisting blood results should be written in the patient notes (*assertion*) - Involving key stakeholders such as resuscitation officers when considering improvements to resuscitation team response (*partnership*)

Each workshop transcript was independently analysed by two members of the research team (JK and SES) to identify examples of approaches for IPC. The examples identified were then classified according to the IPC framework in Table 1, with any new approaches inductively analysed. This combined deductive and inductive approach utilised pre-existing theory, whilst also allowing the theory to be revised for this context, if appropriate [31,35]. JK and SES discussed the approaches using the definitions outlined in Table 1, until agreement on categorisation was reached. Each approach was coded into these larger domains, but also coded textually with a subcategory [31]. Modifications to the initial framework and allocation of subcategories were discussed with the research team, with final decisions made by JK.

Using the revised framework, strategies for IPC were charted according to the subgroup that trainees had described interacting with. A subgroup was defined as a collective group in the workplace that the trainees referred to during the workshop. This included both *intra-*professional colleagues (seniors from the same team, their ward team, other specialties such as surgery or radiology) and *inter-*professional groups (such as nursing or pharmacy). A subgroup labelled ‘general’ was used to index approaches that were not described in relation to one particular group. The use of NVivo (Licence 1.5.2) for data management and indexing of approaches allowed cross-referencing and patterns across the whole dataset to be identified.

### Reflexivity

This is a constructivist study, and our previous clinical, educational, research and personal experiences will have influenced the ideas contained within it. As a senior acute internal medicine specialty trainee, JK was immersed within the topic in her clinical practice and was well situated to interpret and understand the data. SS, as a general practitioner, offered a perspective from another specialty that internal medicine collaborates with. VT, an acute internal medicine consultant, offered views from a more senior clinical position, collaborating with internal medicine trainees on a regular basis. We all have significant experience of medical education research, and our previous research has explored themes such as hierarchy and social identity formation, which will have influenced our research choices and interpretation of findings.

## Results

Seventeen workshops, each two hours in duration, involving 100 trainees, were included in the study. Forty-nine identified as women, 48 identified as men and three trainees chose not to disclose their gender identity. Each domain is discussed with subcategories and illustrative examples below.

### Relationship building

This new domain incorporates ‘Building social capital’ from the original framework, adding other subcategories to better represent the approaches that the trainees took to relationship building.

- ***Building social capital***

Trainees made efforts to build social capital, particularly through acts of kindness towards the ward team or nursing staff:

If you’re going to make a cup of tea, just offer everyone (Trainee 3)

This approach used an element of trade for future gain:

That is a kindness just to make his day a little bit easier by taking something off them and then they’re more likely to be thankful the next time and take something off you (Trainee 9)

Trainees described making the effort to know people’s names. This was evident in general, but especially for their ward team and nursing staff:

Basics, like knowing everyone’s name … it makes it so much easier. When you go ‘Could you just do this, I’m sorry to interrupt you’- it’s awkward. (Trainee 6)

Trainees were clear that building social capital was a way that they could form relationships but also create leverage for when they needed to ask something of others.

- ***Gaining trust***

Trainees aimed to gain trust, especially with seniors and their ward team:

If you’re then demonstrating that you can follow that [ward protocol], and build on that, then that is kind of building on that trust (Trainee 5)

They tried to ‘learn how that department works,’ (Trainee 5) acknowledging that collaboration would be easier going forward, once trust had been established.

- ***Face to face approach***

Trainees described going out of their way to speak face to face with other specialties, particularly radiology. This could be seen as a negotiating tactic, but this also afforded them capital for future discussions:

If you went down and you spoke to them and maybe started recognising your face … it became easier to have a slightly more productive interaction and relationship (Trainee 11)

- ***Civility***

Trainees were advocates of civility in the workplace generally, rather than directed toward one healthcare group in particular:

Civility saves lives … I’d rather fake it a bit and be happier, because it makes for such a nice, informal curricular environment. (Trainee 14)

The various strategies trainees used to build relationships created bonds and a civil workplace environment, but these were also used to build capital and power to ease future collaboration.

### Perspective taking

Trainees reflected on others’ perspectives, particularly of nursing staff and other specialties. This included appreciating others’ workload and others’ priorities.

- ***Appreciating others’ workload***

Internal medicine trainees recognised some of the frustrations that their nursing colleagues may feel and the challenges of the nursing role:

[Nursing is] a lot more physical as well and then I go and sit and write my notes, they [nurses] are running around (Trainee 93)

Internal medicine trainees also considered challenges faced by various surgical specialities, describing perspective taking in practice:

I would hate to have that pager [neurosurgery on call]. It does not stop going off … I would snap. So, I try and remember that when someone’s been not so nice on the end of the phone (Trainee 11)

- ***Appreciating others’ priorities***

Trainees appreciated others’ motives by acknowledging their priorities:

With surgical specialities, their priority is often the patients that they can go do a definitive thing to (Trainee 3)

Trainees recognised that aspects of the nursing role prompted differences in nursing priorities:

Nurses are with the patient 24 hours a day, they’re seeing a lot more, maybe some difficult behaviours. Our priorities are different because we’re getting a snapshot of a patient on ward rounds. (Trainee 55)

Appreciating others’ workload and priorities was something trainees could employ to remain civil in challenging interactions and to understand why some negotiations were difficult.

### Negotiating priorities and resources

The original framework contains both consensus building and influencing strategies. Our data aligned more with the influencing strategies. Reason and assertion, both influencing strategies that were present in the original framework, were not identified within our dataset.

- ***Consensus building***

Regarding other professions, trainees described ‘taking time to explain your rationale’ (Trainee 93). Rather than making demands, they found that collaborating on decision-making was better received:

’Can you give me advice on this?’ And it’s just valuing the knowledge and experience that they’ve got (Trainee 12)

This showed respect but was also a way of creating an illusion of power to obtain the outcome they wanted:

When you’re asking radiology for a scan, instead of asking them for a scan, you sort of word it in a way that you’re asking them for advice on what kind of scan. (Trainee 20)

At times this was genuine, but other examples displayed use of rhetoric suggesting a lack of sincerity:

I always try to approach it like I’m trying to learn from the person, rather than make a referral, because you just avoid all the confrontation … there’s maybe a bit of, like, ego massaging that helps (Trainee 43)

- ***Courting favour***

In addition, trainees found that they could get others onside by being friendly during negotiations:

‘Try to kill them with niceness’ (Trainee 73)

They employed this across all subgroups, describing that its ‘quite disarming’ (Trainee 12):

‘My technique is to double down on being as nice as possible’ (Trainee 15)

- ***Coercion***

Coercion was employed through using the name of a senior, or the threat of a senior becoming involved in the negotiation:

Give the name of the consultant when they feel the referral is not going well (Trainee 46)

Trainees admitted omitting their grade when making telephone referrals:

If you said you were an FY1 [Foundation Year 1 doctor: a newly qualified doctor] you were immediately dismissed and got nowhere. So, you just said, ‘I’m one of the doctors’ (Trainee 22)

They described this as a tactic they had employed since becoming newly qualified doctors and had continued to use this approach.

- ***Partnership***

Trainees described involving the whole team in decision-making and working towards a shared goal:

Everybody buying in to the same idea and going with the same agenda. (Trainee 12)

This approach was mostly used with their ward team, but also with individual members of nursing staff. For example, one trainee described asking a nurse to join them when reviewing a patient:

I will take the person who’s referred them, the nursing staff who’s referred them to me, will come with me … they know them more than you, and if I’m seeing someone on a ward that I’ve never seen before, I want the person who’s looking after them (Trainee 14)

The negotiating tactics described both giving others the illusion of power, for example through ‘ego massaging’ and using power as a threat by involving senior colleagues. Trainees were accustomed to the delicate power imbalances and how to navigate these to succeed in negotiation.

### Shared themes

**Figure 1.**
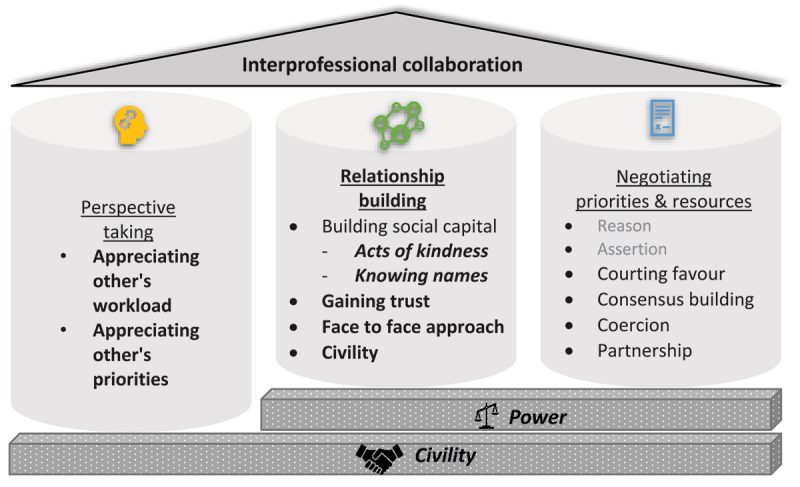
Modified domains of interprofessional collaboration with subcategories of specific strategies. (Modifications to the original framework are in bold with approaches not identified in this context faded. Power and civility are represented as shared themes influencing multiple domains.).

Power and civility were relevant themes across domains. Perspective taking promoted civility. Acts of kindness were performed, and civility upheld to build relationships but also, ultimately, to build social capital and power for future negotiations. When negotiating, courting favour was employed as a tactic, and the power imbalances resulting from medical hierarchy were utilised as a negotiation tool. This is displayed in the revised framework incorporating new and refined codes is illustrated in Figure 1.

### Framework matrix – preventing conflict

Using this revised framework, strategies for IPC were analysed and cross-referenced with various healthcare groups, as summarised in Table 2. The use of framework analysis allowed the visualisation of patterns of approaches utilised by internal medicine trainees. There are obvious gaps within the matrix, particularly with peers and seniors within their own team. Approaches to IPC were most relevant when interacting with other specialties or other professions. For example, with other specialties within the profession, perspective taking and the approaches to negotiation of courting favour and coercion were employed. Perspective taking was also utilised during interactions with colleagues from other professions.

**Table 2.** A multidimensional analysis of approaches to interprofessional collaboration in the workplace and healthcare groups.

Subgroup	Approach to IPC		
	Perspective taking	Relationship building	Negotiating priorities and resources
Seniors		− Gaining trust	
Ward team		− Knowing names − Building social capital − Gaining trust	− Partnership
Other specialties	− Appreciating others’ workload − Appreciating others’ priorities	− Face to face approach	− Courting favour − Coercion − Partnership
Other professions	− Appreciating others’ workload	− Knowing names − Building social capital	− Consensus building − Partnership
General	− Appreciating others’ priorities	− Civility − Building social capital	− Courting favour

The resulting framework matrix demonstrates the ways in which trainees strive to preserve relationships and prevent conflict. Preventing conflict could be achieved through perspective taking, relationship building and effective negotiating. Being civil, whilst tactfully utilising power structures to their advantage, helped trainees when collaborating.

## Discussion

This study investigates the approaches to IPC exercised by internal medicine trainees in the clinical workplace. Bainbridge and Regehr’s framework informed analysis of the data, leading to modifications to the original framework that could be transferable beyond the context of internal medicine. The use of framework analysis allowed appreciation of patterns within the data.

Building and preserving relationships was important for trainees in anticipation of future co-operation and collaboration. Trainees readily expressed the benefits of civility, commenting that ‘civility saves lives’. ‘Civility saves lives’ is a UK-wide campaign promoting civility in healthcare [36] and raising awareness of the far-reaching effects of incivility in the workplace [37–39]. Civility insinuates the peaceful coexistence of diverse social groups and relies on maintaining intergroup empathy and mutual respect [40]. It can refer to verbal and non-verbal communication aimed at or used in the presence of others and through these interactions can contribute to social harmony [40]. It is recognised that civility can be superficial; whilst maintaining peaceful coexistence, it does not necessarily denote a desire for meaningful interaction or relationship [40]. This rhetoric is reflected by trainees who would ‘rather fake it a bit and be happier’. Civility is a prerequisite for the emergence and sustenance of social capital [41] and building relationships. Relationship building is not a static one-off process, but one that requires maintenance over time [23,42]. Given the dynamic and often short-lived nature of teams in modern healthcare, building relationships can be challenging. In this context, adopting a blanket rule for civility, even if relatively superficial at first, is more achievable than relationship building and maintenance. The process of trade of social commodities such as goodwill and respect in collaboration is recognised [43]. Internal medicine trainees demonstrated various ways of forming alliances, creating relationship-based power [44] for future collaboration.

Power imbalances was a recurrent theme, echoing previous work investigating collaboration [45]. In medicine, hierarchy is a rank order of individuals or groups in terms of power and ability to influence [46]. It is often discussed as a negative aspect of the clinical workplace by repressing open communication of low-status individuals [46,47] and contributing to negative experiences of conflict [48]. Hierarchy, and the power differentials that it creates, can be a barrier to IPC, particularly medical dominance over other professions [49–51] prompting calls to flatten hierarchy [52]. Internal medicine trainees described using pre-existing perceptions of power distribution to their advantage, relying on the social order and stability that hierarchies provide [46]. During negotiation, involving a senior with positional power could be used as a threat. Trainees also navigated hierarchy to their advantage by omitting their grade when negotiating on the phone. This was to hide a junior rank in the medical hierarchy, removing positional power from influencing the outcome of their request. Omitting or even lying about one’s grade as a junior doctor has been described as a way around the rules that are created by hierarchy [53]. Trainees’ keen awareness of the influence of power allowed them to exploit it, as has been recognised in newly qualified professionals [45]. Co-operation and negotiation in the workplace are everyday parts of doctors’ jobs. With power being a major influence; it must be considered and included when implementing training for collaboration.

### Research and practical implications

Bringing these domains of IPC to the attention of students and practitioners could help them to make sense of intergroup challenges in the workplace [23]. For example, the uni-professional, uni-disciplinary workshop as described in this study, when underpinned by the domains outlined, could be a useful opportunity to outline demonstrable approaches and be explicit about the influence of power differentials [48]. Although conflict management education is advocated within health professional curricula [10,13,54–57], training for IPC training might better focus on conflict prevention. For example, training in negotiation strategies for healthcare professionals has been recommended [45]. Consensus building and partnership through shared goals could be targeted and promoted through educational intervention. Having a shared purpose is a recognised mechanism for IPC, although its outcomes are variable and it requires further study [58]. Perspective taking can lead to increased empathy and decreased prejudice against out-groups [59] as well as potentially mitigating moral distress [60]. Given the propensity for the trainees in this study to use this approach in clinical practice, perspective taking is a realistic strategy to actively encourage within training. Promoting civility includes an intolerance of incivility in the workplace where persistent incivility is penalised [37] and is an area which warrants further study. We have summarised key considerations moving forward in Table 3.

**Table 3.** Key considerations for educators and healthcare leaders.

Key considerations going forward:
Promote perspective taking of others’ workload and priorities
Promote civility in the workplace
Consider negotiation training for healthcare professionals
Acknowledge power imbalances within interventions for IPC

### Strengths and limitations

This national study explores the lived experiences of trainees, allowing their narratives to inform understanding and the development of tailored educational interventions. The uni-professional nature of the data collection can be seen as both a limitation and a strength. There is a recognised need to train for collaboration through developing individual awareness and skills as antecedent to improved team work [23]. It could also be argued that trainees are more likely to express honest thoughts and feedback in a uni-professional context about challenges they have with IPC and the strategies they employ.

Given the use of a theoretical framework, there may be some transferability of the results and strategies to other groups. It must be acknowledged that the examples of approaches to collaboration are what trainees *say* they do, which may differ from how they *actually* behave in the clinical environment. It is also possible that the results are skewed towards memorable or difficult interactions.

It is recognised that challenging interprofessional communication might be a difficult topic for some to discuss. The workshop setting may have inhibitive effects on voicing to a group, but may also encourage the disclosure of similar experiences. The facilitators were senior doctors, and therefore their professional position may have influenced trainees’ willingness to share experiences and preconceptions. The facilitators aimed to create a psychologically safe environment with lack of judgement and clear delineation that the trainees’ reflections would be heard in confidence and deidentified.

## Conclusions

This framework analysis study exhibits the strategies for IPC described by internal medicine trainees in the context of interaction with various healthcare groups. The themes of power and civility transcended multiple domains, highlighting the impact of both. This study presents a modified framework for IPC, with specific strategies therein, which could inform educational interventions for collaboration. The findings should be of interest to those striving to create a collaborative healthcare workplace.
